# Prognostic significance of miR-122 expression after curative resection in patients with hepatocellular carcinoma

**DOI:** 10.1038/s41598-019-50594-2

**Published:** 2019-10-14

**Authors:** Sang Yun Ha, Jeong Il Yu, Changhoon Choi, So Young Kang, Jae-Won Joh, Seung Woon Paik, Seonwoo Kim, Minji Kim, Hee Chul Park, Cheol-Keun Park

**Affiliations:** 1 Department of Pathology and Translational Genomics, Samsung Medical Center, Sungkyunkwan University School of Medicine, Seoul, Korea; 2 Department of Radiation Oncology, Samsung Medical Center, Sungkyunkwan University School of Medicine, Seoul, Korea; 3 Department of Surgery, Samsung Medical Center, Sungkyunkwan University School of Medicine, Seoul, Korea; 4 Department of Internal Medicine, Samsung Medical Center, Sungkyunkwan University School of Medicine, Seoul, Korea; 5 Statistics and Data Center, Samsung Medical Center, Sungkyunkwan University School of Medicine, Seoul, Korea; 6 Department of Pathology, Anatomic Pathology Reference Lab, Seegene Medical Foundation, Seoul, Korea

**Keywords:** Prognostic markers, Hepatocellular carcinoma

## Abstract

Downregulation of MicroRNA-122 (miR-122) and its association with cancer progression have been reported in hepatocellular carcinoma (HCC) cell line models and a limited number of HCC samples. Recently, restoration of miR-122 expression by direct delivery of miR-122 yielded promising results in HCCs. However, the prognostic effect of miR-122 expression in human HCC samples is not fully understood. We investigated the expression level of miR-122 by quantitative real-time polymerase chain reaction in 289 curatively resected HCC samples and 20 normal liver samples and evaluated the prognostic effect of miR-122 expression. The relative quantification value of miR-122 was much lower in HCC samples than in normal liver tissues. During a median 119 months of follow-up for survival, the low miR-122 expression group showed shorter recurrence-free survival (RFS) (p = 0.033) and intrahepatic recurrence-free survival (IHRFS) (p = 0.014), and a trend of short distant metastasis-free survival (DMFS) (p = 0.149) than high expression group. On multivariate analysis, miR-122 expression was an independent prognostic factor for RFS, IHRFS and DMFS. Downregulation of miR-122 expression, frequently found in HCC samples, was an independent prognostic factor for RFS after curative resection. Emerging therapeutic approaches targeting miR-122 could be applicable in patients with miR-122 downregulated hepatocellular carcinoma.

## Introduction

The overall survival rate of patients with hepatocellular carcinoma (HCC) remains unsatisfactory despite improvements in surveillance and clinical treatment strategies because of frequent recurrence and metastasis even after hepatic resection and lack of effective adjuvant therapy^1,2^. Identification of new therapeutic targets or reliable biomarkers is needed to ensure more effective clinical treatment after curative resection^3^.

MicroRNA-122 (miR-122) is the most abundant liver-specific miRNA, and its expression is conserved in all vertebrates, reflecting its critical role^4–7^. It is well known that miR-122 is involved in lipid metabolism, iron homeostasis, and differentiation of hepatocytes^8–12^. Also, expression of miR-122 has been reported to be downregulated in HCCs^13–20^, and its role as a tumor suppressant in hepatocellular carcinogenesis has been shown using *in vitro* and *in vivo* experimental models^10,21,22^. A few previous studies showed shorter survival time in patients with HCC with low miR-122 expression^23–26^. However, the clinical role of miR-122 expression in human HCC samples is not fully understood.

Based on evidence of the tumor suppressive effect of miR-122 in HCCs, direct delivery of miRNA oligonucleotides into tumors for restoration of miR-122 expression has been performed as a therapeutic strategy against HCC with promising results^22,27–29^. Given the potential clinical application of miR-122 restoration in the treatment of HCCs, it is necessary to evaluate miR-122 expression and its clinical role in HCC samples.

In this study, we examined miR-122 expression by quantitative Reverse-Transcriptase Polymerase Chain Reaction (qRT-PCR) in 289 HCC samples and analyzed the prognostic effect of miR-122 expression in HCC patients with a long-term follow up period (median follow-up 119 months for survival).

## Results

### Patients

The characteristics of patients and tumors at the time of surgery are summarized in Table 1

**Table 1:** Characteristics of patients and tumors, at the time of surgery. ALBI, Albumin-Bilirubin; AFP, α-fetoprotein; AJCC, American Joint Committee on Cancer; BCLC, Barcelona Clinic Liver Cancer; ^a)^Partial data was not available.

Characteristics		No. of patients (%)
Age	Median (range)	53 (17–76)
Sex	Female	51 (17.6)
	Male	238 (82.4)
Etiology	Hepatitis B virus	220 (76.1)
	Hepatitis C virus	25 (8.6)
	Hepatitis B virus and Hepatitic C virus	4 (1.4)
	Alcohol	18 (6.2)
	Others	22 (7.6)
Child-Pugh Class	A	288 (99.7)
	B	1 (0.3)
Albumin level, g/dL	Median (range)	4.0 (2.8–5.0)
	>3.5	260 (90.0)
	≤3.5	29 (10.0)
ALBI grade	2	93 (32.2)
	1	196 (67.8)
AFP level, ng/mL^a)^	Median (range)	169.5 (1.0–1667054.0)
	≤200	175 (62.7)
	>200	104 (37.3)
AJCC T-stage	1	122 (42.2)
	2	116 (40.1)
	3A	33 (11.4)
	3B	12 (4.2)
	4	6 (2.1)
BCLC stage	0	2 (0.7)
	A1	142 (49.1)
	A2	5 (1.7)
	A4	16 (5.5)
	B	109 (37.7)
	C	15 (5.2)
Tumor size, cm	Median (range)	3.7 (1.0–21.0)
	≤5.0	191 (66.1)
	>5.0	98 (33.9)
Edmondson grade	I	32 (11.1)
	II	233 (80.6)
	III	24 (8.3)
Microvascular invasion	Yes	159 (55.0)
	No	130 (45.0)
Major portal vein invasion	Yes	13 (4.5)
	No	276 (95.5)
Intrahepatic metastasis	Yes	68 (23.5)
	No	221 (76.5)
Multicentric occurrence	Yes	19 (6.6)
	No	270 (93.4)
Backgound liver tissue	Cirrhosis	145 (50.2)
	Chronic active hepatitis	67 (23.2)
	Chronic persistent hepatitis	44 (15.2)
	Alcoholic hepatitis	15 (5.2)
	Reactive hepatitis	13 (4.5)
	Others	5 (1.7)

. The median age was 53 years (range, 17–76). Out of 289 patients, 220 (76.1%) showed HCC related to hepatitis B virus (HBV), and 29 (10.0%) showed HCC related to hepatitis C virus (HCV). Approximately 80% of patients had American Joint Committee on Cancer (AJCC) T stage 1 or 2. Median tumor size was 3.7 cm, and about one-third of the patients had a tumor with larger than 5 cm. Microvascular invasion, major portal invasion, intrahepatic metastasis, and multicentric occurrence was observed in 55.0%, 4.5%, 23.5%, and 6.6% of patients, respectively. Approximately 50% of HCCs occurred in the background of cirrhosis.

### miR-122 expression in HCC tissues and its association with clinicopathologic parameters

The assay showed a linear dynamic range of log concentration, and an efficiency of 97% (Supplementary Fig. S1). The intra- and inter-assay coefficient of variabilities ranged from 0.3% to 1.8% and 0.8% to 1.7%, respectively.

**Figure 1:**
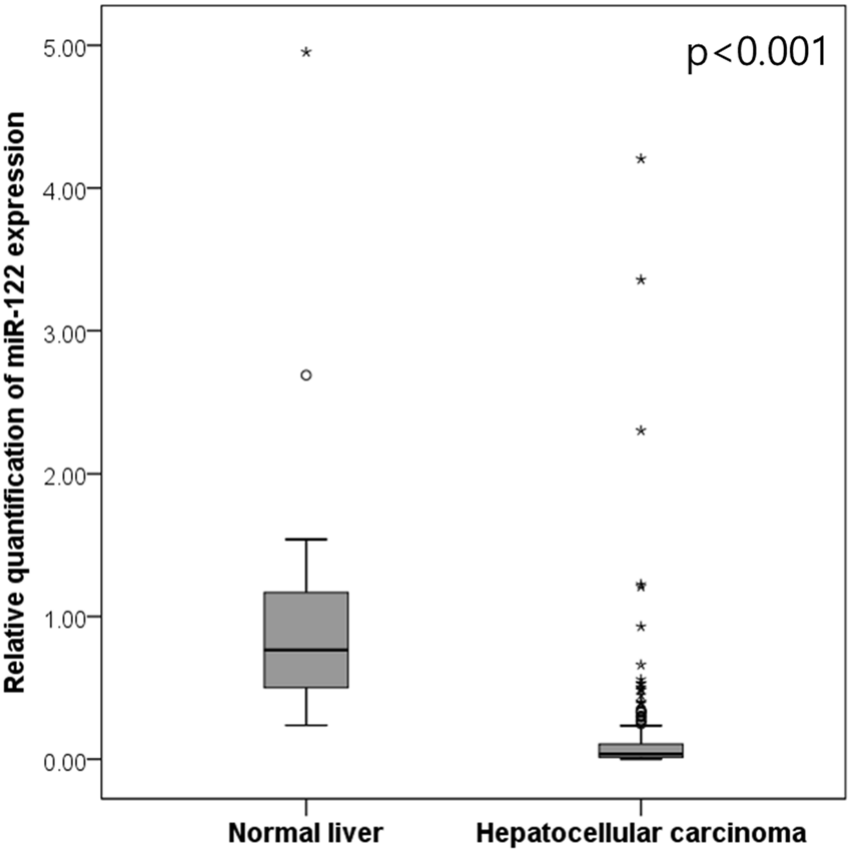
The relative quantification value of miR-122 in hepatocellular carcinoma and normal liver.

The relative quantification (RQ) value of miR-122 was significantly lower in HCC samples than in normal liver tissues (mean ± standard deviation: 0.12 ± 0.37 vs. 1.07 ± 1.07, p < 0.001 by Mann- Whitney U test) (Fig. 1). About 90% of the HCC samples showed a value lower than the minimum value (0.24) of normal liver tissues, whereas only five HCC cases showed a value higher than the mean value (1.07) of normal liver tissues.

The RQ value of miR-122 showed a tendency to be lower in large sized (>5 cm) HCCs compared with small (≤5 cm) HCCs (mean 0.073 ± 0.100 vs. 0.150 ± 0.445) and in BCLC (Barcelona Clinic Liver Cancer) stage B,C compared with BCLC stage 0,A (mean 0.076 ± 0.098 vs. 0.159 ± 0.477), but these differences did not reach statistical significance by Mann Whitney test. The RQ value of miR-122 in HCC with other prognostic factors such as advanced AJCC T stage, intrahepatic metastasis, microvascular invasion, or Edmondson grade III or IV was slightly lower than in other groups, but failed to demonstrate statistical significance (data not shown).

The median of RQ value in 289 HCC samples was 0.036 (range, 0.000–4.205). The estimated cutoff value of miR-122 with the highest level of statistical significance related to Recurrence free survival (RFS) was 0.17 by the minimum p-value approach. This cutoff value was internally validated using 1,000 bootstrap samples generated from the study data with replacement. The estimated cutoff value of 0.17 was located between 0.007 and 0.400, which were the lower and upper 5th percentiles of the estimates, respectively. Out of 289 cases, 44 (15.2%) were classified into the high expression group and 245 (84.8%) into the low expression group. The associations between miR-122 expression and clinicopathologic parameters are summarized in Table 2. miR-122 expression was not significantly associated with any clinicopathologic factor including well-known prognostic factors of HCC. Clinical information and RQ value of miR-122 in each sample are provided in Supplementary Dataset 1.

**Table 2:** The association between miR122 expression and clinicopathologic parameters.

	Total	miR122 expression		p value
		Low (RQ value ≤ 0.17)	High (RQ value > 0.17)	
		n = 245 (24.8%)	n = 44 (15.2%)	
**Age, year**				
≤55	167	143 (58.4%)	24 (54.5%)	0.636
>55	122	102 (41.6%)	20 (45.5%)	
**Gender**				
Female	51	45 (18.4%)	6 (13.6%)	0.448
Male	238	200 (81.6%)	38 (86.4%)	
**Tumor size, cm**				
≤5.0	191	160 (65.3%)	31 (70.5%)	0.507
>5.0	98	85 (34.7%)	13 (29.5%)	
**Edmondson grade**				
I	32	27 (11.0%)	5 (11.4%)	0.617^c)^
II	233	199 (81.2%)	34 (77.3%)	
III	24	19 (7.8%)	5 (11.4%)	
**Microvascular invasion**				
(−)	130	110 (44.9%)	20 (45.5%)	0.946
(+)	159	135 (55.1%)	24 (54.5%)	
**Major portal vein invasion**				
(−)	276	233 (95.1%)	43 (97.7%)	0.439
(+)	13	12 (4.9%)	1 (2.3%)	
**Intrahepatic metastasis**				
(−)	221	187 (76.3%)	34 (77.3%)	0.892
(+)	68	58 (23.7%)	10 (22.8%)	
**Multicentric occurrence**				
(−)	270	230 (93.9%)	40 (90.1%)	0.506^c)^
(+)	19	15 (6.1%)	4 (9.9%)	
**AJCC T-stage**				
1	122	105 (42.9%)	17 (38.6%)	0.942^c)^
2	116	97 (39.6%)	19 (43.2%)	
3	45	38 (18.4%)	7 (15.9%)	
4	6	5 (2.0%)	1 (2.3%)	
**BCLC stage**				
0-A	165	138 (56.3%)	27 (61.4%)	0.887^c)^
B	109	94 (44.5%)	15 (34.1%)	
C	15	13 (5.3%)	2 (4.5%)	
**Albumin level, g/dL**				
>3.5	260	218 (89.0%)	42 (93.3%)	0.276^c)^
≤3.5	29	27 (11.0%)	2 (6.7%)	
**AFP level, ng/mL** ^**a)**^				
≤200	175	146 (61.1%)	29 (72.5%)	0.167
>200	104	93 (38.9%)	11 (27.5%)	
**Etiology**				
Non-viral	40	33 (16.3%)	7 (15.9%)	0.567^c)^
HBV	220	186 (89.8%)	34 (77.3%)	
HCV	25	23 (9.4%)	2 (4.5%)	
HBV and HCV	4	3 (1.2%)	1 (2.3%)	
**Liver cirrhosis**				
(−)	144	125 (51.0%)	19 (43.2%)	0.338
(+)	145	120 (49.0%)	25 (56.8%)	
**Early intrahepatic recurrence**				
(≤2 years)				
(−)^b)^	164	134 (54.7%)	30 (68.2%)	0.096
(+)	125	111 (45.3%)	14 (31.8%)	
**Late intrahepatic recurrence**				
(>2 years)				
(−)^b)^	85	65 (55.1%)	20 (76.9%)	0.040
(+)	59	53 (44.9%)	6 (23.1%)	
**Distant metastasis**				
(−)	194	160 (65.3%)	34 (77.3%)	0.120
(+)	95	85 (34.7%)	10 (22.7%)	

AJCC, American Joint Committee on Cancer; BCLC, Barcelona Clinic Liver Cancer;

AFP, α-fetoprotein; HBV, hepatitis B virus; HCV, hepatitis C virus.

^a)^Partial data was not available ^b)^No early or late recurrence, ^c)^by Fisher’s exact test, otherwise by chi-square test.

### Recurrence pattern and predictive factors for early and late intrahepatic recurrence

During the median 120 months (range 2–193) of follow-up, recurrence was detected in 197 patients (68.2%). Among them, 82 patients (28.4%) had both intrahepatic recurrence (IHR) and distant metastasis (DM), 102 patients (35.3%) had IHR only, and 13 patients (4.5%) had DM only. Among all patients with IHR (with or without DM), early IHR developed in 125 (43.4%) patients, and late IHR was detected in 59 of the 145 patients (40.7%) that were followed for over 2 years.

The results of univariate and multivariate analyses of clinicopathologic parameters and early or late IHR are summarized in Tables 3 and 4. In multivariate analysis, intrahepatic metastasis (HR 8.29 [95% CI 2.28–30.17], p = 0.001 by the Cox proportional hazard model), Albumin-Bilirubin (ALBI) grade (HR 2.13 [95% CI 1.10–4.13], p = 0.026 by the Cox proportional hazard model), and liver cirrhosis (HR 2.01 [95% CI 1.08–3.72], p = 0.028 by the Cox proportional hazard model) were independent predictive factors for early IHR. miR-122 expression showed marginal significance for predicting early IHR (HR 2.02 [95% CI 0.91–4.47], p = 0.083 by the Cox proportional hazard model).

**Table 3:** Univariate and multivariate analyses for predicting early intrahepatic recurrence. AJCC, American Joint Committee on Cancer; BCLC, Barcelona Clinic Liver Cancer; ALBI, Albumin-Bilirubin; AFP, α-fetoprotein.

	Univariate		Multivariate	
	HR (95% CI)	p value	HR (95% CI)	p value
**Age, year**				
>55 vs ≤55	1.01 (0.63–1.62)	0.956		
**Gender**				
Male vs Female	1.11 (0.60–2.05)	0.742		
**Tumor size, cm**				
>5.0 vs ≤5.0	1.95 (1.19–3.19)	0.008	0.76 (0.21–2.77)	0.681
**Edmondson grade**				
III vs I, II	1.94 (0.83–4.53)	0.125	1.16 (0.42–3.20)	0.78
**Microvascular invasion**				
(+) vs (−)	3.12 (1.90–5.10)	<0.001	1.49 (0.77–2.88)	0.238
**Major portal vein invasion**				
(+)vs (−)	4.67 (1.26–17.33)	0.021	0.57 (0.16–2.86)	0.498
**Intrahepatic metastasis**				
(+)vs (−)	10.28 (5.18–20.40)	<0.001	8.29 (2.28–30.17)	0.001
**Multicentric occurrence**				
(+)vs (−)	0.95 (0.37–2.44)	0.917		
**AJCC T-stage**				
3,4 vs 1,2	5.74 (2.86–11.56)	<0.001	1.08 (0.25–4.67)	0.924
**BCLC stage**				
B,C vs 0, A	2.93 (1.80–4.75)	<0.001	1.48 (0.44–5.01)	0.527
**Albumin level, g/dL**				
≤3.5 vs >3.5	1.99 (0.92–4.35)	0.083	0.97 (0.35–2.66)	0.948
**ALBI grade**				
2 vs 1	2.79 (1.68–4.63)	<0.001	2.13 (1.10–4.13)	0.026
**AFP level, ng/mL**				
>200 vs ≤200	1.36 (0.83–2.21)	0.222		
**Etiology**				
Viral vs Non-viral	2.23 (1.07–4.65)	0.033	1.13 (0.48–2.67)	0.777
**Liver cirrhosis**				
(+)vs (−)	1.79 (1.12–2.87)	0.015	2.01 (1.08–3.72)	0.028
**miR122 expression**				
Low vs High	1.78 (0.90–3.51)	0.099	2.02 (0.91–4.47)	0.083

**Table 4:** Univariate and multivariate analyses for predicting late intrahepatic recurrence. AJCC, American Joint Committee on Cancer; BCLC, Barcelona Clinic Liver Cancer; ALBI, Albumin-Bilirubin; AFP, α-fetoprotein.

	Univariate		Multivariate	
	HR (95% CI)	p value	HR (95% CI)	p value
**Age, year**				
>55 vs ≤55	1.08 (0.55–2.13)	0.823		
**Gender**				
Male vs Female	1.23 (0.52–2.90)	0.645		
**Tumor size, cm**				
>5.0 vs ≤5.0	0.52 (0.24–1.13)	0.100	0.59 (0.24–1.41)	0.232
**Edmondson grade**				
III vs I, II	0.71 (0.17–2.94)	0.632		
**Microvascular invasion**				
(+)vs (−)	0.74 (0.37–1.45)	0.375		
**Major portal vein invasion**				
(+)vs (−)	NA			
**Intrahepatic metastasis**				
(+)vs (−)	1.09 (0.23–5.04)	0.917		
**Multicentric occurrence**				
(+)vs (−)	6.11 (0.67–56.11)	0.110	4.08 (0.42–39.65)	0.225
**AJCC T-stage**				
3,4 vs 1,2	0.86 (0.20–3.73)	0.837		
**BCLC stage** ^**a)**^				
B,C vs 0, A	0.52 (0.24–1.11)	0.090		
**Albumin level, g/dL**				
≤3.5 vs >3.5	0.68 (0.16–2.83)	0.595		
**ALBI grade**				
2 vs 1	1.22 (0.54–2.77)	0.637		
**AFP level, ng/mL**				
>200 vs ≤200	1.84 (0.91–3.75)	0.092	1.86 (0.88–3.95)	0.107
**Etiology**				
Viral vs Non-viral	2.72 (1.02–7.26)	0.046	1.86 (0.65–5.33)	0.246
**Liver cirrhosis**				
(+)vs (−)	1.75 (0.89–3.42)	0.104	1.43 (0.66–3.10)	0.366
**miR122 expression**				
Low vs High	2.72 (1.02–7.26)	0.046	3.04 (1.02–9.06)	0.046

^a)^Not included in multivariable analysis due to multicollinearity with tumor size.

For predicting late IHR, etiology and miR-122 expression were significant factors in univariate analysis, and miR-122 expression was the only significant factor in multivariate analysis (HR 3.04 [95% CI 1.02–9.06], p = 0.046 by the Cox proportional hazard model).

### Impact of miR-122 expression on the recurrence-free survival of HCC patients

**Figure 2:**
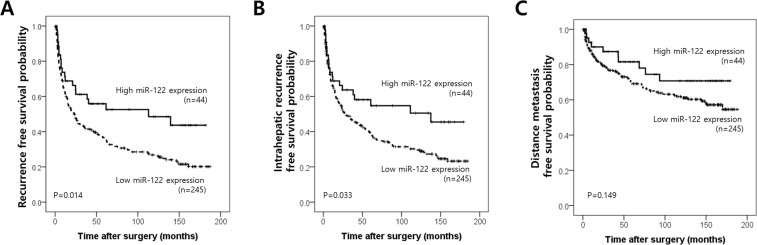
Kaplan Meier survival curves of recurrence free survival (**A**), intrahepatic recurrence free survival (**B**) and distant metastasis free survival (**C**) according to miR-122 expression status.

Patients with low miR-122 expression showed shorter IHRFS and RFS rates than those with high miR-122 expression (p = 0.014 and p = 0.033 by log-rank test, respectively) and a trend toward a shorter DMFS rate (p = 0.149 by log-rank test) (Fig. 2). Results of univariate analysis of probable prognostic factors for intrahepatic RFS, DMFS, and RFS are summarized in Table 5. In multivariate analyses, significant prognostic factors were intrahepatic metastasis, multicentric occurrence, ALBI grade, and miR-122 expression for intrahepatic RFS; Edmondson grade, intrahepatic metastasis, and miR-122 expression for DMFS; and intrahepatic metastasis, ALBI grade, and MiR-122 expression for RFS (Table 6). In addition to intrahepatic metastasis, miR-122 expression was an independent prognostic factor for IHRFS (HR 1.89 [95% CI 1.16–3.07], p = 0.010 by the time-dependent Cox model), DMFS (HR 2.14 [95% CI 1.05–4.36], p = 0.036 by the time-dependent Cox model), and RFS (HR 2.17 [95% CI 1.34–3.52], p = 0.002 by the time-dependent Cox model).

**Table 5:** Univariate analysis of probable prognostic factors in intrahepatic recurrence- free survival, distant metastasis- free survival, and recurrence free survival. AJCC, American Joint Committee on Cancer; BCLC, Barcelona Clinic Liver Cancer; ALBI, Albumin-Bilirubin; AFP, α-fetoprotein.

	Intrahepatic recurrence free survival		Distant metastasis free survival		Recurrence free survival	
	HR (95% CI)	p value	HR (95% CI)	p value	HR (95% CI)	p value
**Age, year**						
>55 vs ≤55	1.04 (0.78–1.40)	0.791	1.00 (0.67–1.51)	0.995	0.96 (0.72–1.27)	0.756
**Gender**						
Male vs Female	1.15 (0.78–1.70)	0.469	0.81 (0.50–1.30)	0.38	1.06 (0.74–1.53)	0.754
**Tumor size, cm**						
>5.0 vs ≤5.0	1.43 (1.05–1.93)	0.022	2.37 (1.58–3.55)	<0.001	1.68 (1.26–2.24)	<0.001
**Edmondson grade**						
III vs I, II	1.51 (0.92–2.49)	0.107	2.88 (1.63–5.09)	<0.001	1.81 (1.13–2.90)	0.014
**Microvascular invasion**						
(+)vs (−)	1.86 (1.39–2.50)	<0.001	2.85 (1.83–4.44)	<0.001	2.14 (1.60–2.86)	<0.001
**Major portal vein invasion**						
(+)vs (−)	3.31 (1.74–6.29)	<0.001	6.02 (3.09–11.73)	<0.001	3.43 (1.86–6.33)	<0.001
**Intrahepatic metastasis**						
(+)vs (−)	4.67 (3.37–6.47)	<0.001	4.76 (3.12–7.28)	<0.001	4.58 (3.34–6.29)	<0.001
**Multicentric occurrence**						
(+)vs (−)	1.57 (0.87–2.82)	0.136	1.17 (0.51–2.67)	0.713	1.39 (0.78–2.51)	0.268
**AJCC T-stage**						
3,4 vs 1,2	3.21 (2.26–4.57)	<0.001	4.57 (2.94–7.12)	<0.001	3.84 (2.73–5.39)	<0.001
**BCLC stage**						
B,C vs 0, A	1.82 (1.36–2.43)	<0.001	2.74 (1.82–4.12)	<0.001	2.05 (1.54–2.71)	<0.001
**Albumin level, g/dL**						
≤3.5 vs>3.5	1.92 (1.21–3.03)	0.005	1.80 (0.98–3.30)	0.058	1.84 (1.18–2.87)	0.008
**ALBI grade**						
2 vs 1	1.82 (1.35–2.47)	<0.001	1.60 (1.06-2.43)	0.026	1.85 (1.38–2.47)	<0.001
**AFP level, ng/mL**						
>200 vs ≤200	1.42 (1.05–1.91)	0.023	1.76 (1.17–2.66)	0.007	1.62 (1.21–2.16)	0.001
**Etiology**						
Viral vs Non-viral	1.90 (1.15–3.13)	0.012	1.60 (0.80–3.17)	0.182	1.88 (1.17–3.02)	0.009
**Liver cirrhosis**						
(+)vs (−)	1.43 (1.07–1.92)	0.015	1.05 (0.70–1.57)	0.826	1.34 (1.02–1.78)	0.039
**miR122 expression**						
Low vs High	1.65 (1.04–2.63)	0.035	1.61 (0.84–3.11)	0.153	1.75 (1.11–2.75)	0.016

**Table 6:** Multivariate analysis of probable prognostic factors in intrahepatic recurrence- free survival, distant metastasis- free survival, and recurrence free survival. AJCC, American Joint Committee on Cancer; BCLC, Barcelona Clinic Liver Cancer; ALBI, Albumin-Bilirubin; AFP, α-fetoprotein.

	Intrahepatic recurrence free survival		Distant metastasis free survival		Recurrence free survival	
	HR (95% CI)	p value	HR (95% CI)	p value	HR (95% CI)	p value
**Tumor size, cm**						
>5.0 vs ≤5.0	0.97 (0.44–2.13)	0.935	1.05 (0.39–2.83)	0.929	0.94 (0.60–1.95)	0.788
**Edmondson grade**						
III vs I, II	1.12 (0.6401.94)	0.699	2.47 (1.34–4.56)	0.004	1.22 (0.73–2.04)	0.452
**Microvascular invasion**						
(+)vs (−)	1.12 (0.77–1.63)	0.548	1.55 (0.90–2.67)	0.111	1.42 (0.10–2.02)	0.051
**Major portal vein invasion**						
(+)vs (−)	0.90 (0.44–1.81)	0.760	1.73 (0.81–3.68)	0.156	0.76 (0.37–1.55)	0.758
**Intrahepatic metastasis**						
(+)vs (−)	5.21 (3.68–7.38)	<0.001	4.78 (3.08–7.41)	<0.001	3.97 (2.69–5.85)	<0.001
**Multicentric occurrence**						
(+)vs (−)	1.94 (1.03–3.62)	0.039			1.54 (0.82–2.90)	0.183
**AJCC T-stage**						
3,4 vs 1,2	1.03 (0.58–1.82)	0.918	1.39 (0.62–3.13)	0.423	1.49 (0.86–2.60)	0.156
**BCLC stage**						
B,C vs 0, A	0.90 (0.60–1.36)	0.622	1.11 (0.62–1.98)	0.729	1.16 (0.56–2.40)	0.691
**Albumin level, g/dL**						
≤3.5 vs>3.5	1.32 (0.77–2.23)	0.311	1.42 (0.70–2.87)	0.335	1.30 (0.77–2.20)	0.32
**ALBI grade**						
2 vs 1	1.41 (1.02–1.94)	0.038	1.41 (0.92–2.16)	0.117	1.47 (1.08–2.02)	0.015
**AFP level, ng/mL**						
>200 vs ≤200	1.08 (0.79–1.49)	0.628	1.10 (0.70–1.73)	0.688	1.19 (0.88–1.62)	0.26
**Etiology**						
Viral vs Non-viral	1.01 (0.86–1.20)	0.869	0.92 (0.72–1.16)	0.477	0.99 (0.84–1.65)	0.887
**Liver cirrhosis**						
(+) vs (−)	1.32 (0.97–1.79)	0.078			1.29 (0.96–1.74)	0.096
**miR122 expression**						
Low vs High	1.89 (1.16–3.07)	0.010	2.14 (1.05–4.36)	0.036	2.17 (1.34–3.52)	0.002

## Discussion

In this study, we demonstrated that miR-122 expression was downregulated in 289 HCC samples compared with normal liver tissues and was an independent predictive factor for late IHR as well as an independent prognostic factor for RFS regardless of recurrence pattern (intrahepatic recurrence or distant metastasis) in a large cohort of HCC patients with long-term follow-up.

There have been numerous reports on the role of miR-122 as a tumor suppressor in hepatocarcinogenesis, performed at various confidence levels. Downregulation of miR-122 in HCCs has been reported in several studies^13–20^, leading to further *in vitro* and *in vivo* studies. Tsai *et al*. showed that miR-122 was significantly downregulated in T3 HCCs with intrahepatic metastasis compared with T1 HCCs or adjacent normal liver tissue, and that restoration of miR-122 reduced *in vitro* migration, invasion, and anchorage-independent growth as well as tumorigenesis *in vivo* in a nude mouse xenograft model^21^. In 2012, Hsu *et al*. and Tsai *et al*. provided the strongest evidence for an antitumor function of miR-122 in liver. In their studies, mice with germline knockout or liver-specific knockout of miR-122 developed spontaneous HCCs in addition to steatohepatitis and fibrosis, and restoration of miR-122 reduced tumor development^10,22^. However, the clinical effect of miR-122 expression in HCC patients, especially on patient survival, has not been fully elucidated. Coulouarn *et al*. reported that patients with low miR-122 expression showed shorter overall survival times than those with high miR-122 expression (30.3 ± 8.0 months vs. 83.7 ± 10.3 months, p < 0.001) in 64 HCC patients^26^. They also showed that miR-122 expression was low in HCCs with poor differentiation, high proliferation, low apoptotic index, and large tumor size^26^. In a recent meta-analysis by Zhang *et al*. incorporating of 4 studies containing 328 patients, low miR-122 expression in tissue or fine needle aspiration sample was significantly associated with unfavorable overall survival and progression free survival. In validation using TCGA dataset, low miR-122 expression was significantly associated with OS and marginally associated with PFS^30^. Also, significance of serum miR-122 expression in HCC have been reported. Qiao *et al*. showed that serum miR-122 expression was significantly lower in HBV-related HCC patients than in benign liver disease group or control group^31^. A few recent reports suggested serum exosomal miR-122 as a predictive biomarker in transarterial chemoembolization-treated HCC patients or diagnostic biomarkers for HCC^32,33^.

In this study, we showed that miR-122 expression was downregulated in 289 HCC samples compared with normal liver tissues. About 90% of the HCC samples showed a value lower than the minimum expression value of normal liver tissues. Also, we demonstrated that miR-122 expression was an independent prognostic factor for RFS in a large cohort of 289 HCC patients with long-term follow-up. These results are consistent with results from previous studies and provide clinical evidence suggesting miR-122 as a potential therapeutic tool targeting HCC. Interestingly, survival curves are clearly distinguished after 2 years from surgery, as depicted in survival curve of Fig. 2, and low expression of miR-122 was associated with frequent late intrahepatic recurrence, which could represent a de novo HCC, not metastasis from primary tumor. Based on these results, we can suggest that patients with HCC having low expression of miR-122 may require intensive follow-up even after 2 years from surgery. These results are somewhat different from previous studies showing association between low expression of miR-122 and aggressive tumor behavior which represents early intrahepatic recurrence^22,26,34^. This study includes HCCs with curative resection, which had no known metastasis at the time of surgery. We observed that miR-122 expression is slightly lower in HCCs with factors associated with tumor aggressiveness, such as larger size (>5 cm), intrahepatic metastasis, higher Edmondson grade, or microvascular invasion, but it did not reach the statistical significance. The effect could be less obvious because of the relatively uniform study population. Meanwhile, miR-122 down-regulation is known to be involved in the progression of liver fibrosis and emergence of de novo HCC^10^. It can be inferred that de-novo HCCs by down-regulation of miR-122 could have low expression of miR-122. There have been only 4 studies regarding prognostic effect of miR-122 expression in HCC tissue samples, and the largest patient number was 144^23–26^. Further study is necessary for validating the prognostic effect of miR-122 expression.

Based on previous studies supporting a tumor suppressive effect of miR-122 in HCCs, restoration of miR-122 expression represents an interesting strategy for the treatment of HCC. Direct delivery of miRNA mimic oligonucleotides into tumors by viral vectors such as the adeno-associated virus 8 (AAV8) serotype or LNP-DP1, a cationic lipid- based nanoparticle, has been tested with promising results^22,27–29^. It is noteworthy that the first miRNA mimic, MRX34, a liposomal miRNA 34 mimic, is now undergoing testing in a multicenter phase I study in a variety of cancers including HCCs (ClinicalTrials.gov.identifier: NCT01829971A)^35^. In addition, miR-122 has therapeutic applications in the aspect of sensitization of chemotherapy. It has been reported that restoration of miR-122 sensitizes HCCs to chemotherapeutic agents such as sorafenib, doxorubicin, vincristine, and cisplatin^36–39^. A combination of miR-122 restoration and chemotherapy could be another therapeutic strategy against HCC.

This study has some limitations. First, this study was retrospectively performed in a single institution and included patients who had undergone curative resection for primary tumor, thus possibly introducing unavoidable selection bias. Consequently, our results should be interpreted cautiously, and further study with a large population and prospective design is needed. Second, the estimated cutoff value of miR-122 expression was not validated in an independent cohort. We redeemed this by internal validation using 1,000 bootstrap samples generated from the study data with replacement, but further external validation would be required.

Despite these limitations, our study provides valuable information confirming the prognostic significance of miR-122 expression in the largest cohort of HCC patients with long-term follow-up. Our data showed that miR-122 is an independent predictive factor for late IHR and an independent prognostic factor for RFS regardless of recurrence pattern, either intrahepatic recurrence or distant metastasis.

In conclusion, miR-122 expression is frequently downregulated in HCCs, and miR-122 expression is an independent prognostic factor for recurrence-free survival after curative resection. Emerging therapeutic approaches targeting miR-122 could be applicable in patients with miR-122-downregulated hepatocellular carcinoma.

## Material and Methods

### Patients and samples

Initially, a total of 291 patients who underwent curative resection for primary HCC at Samsung Medical Center, Seoul, Korea, from July 2000 to May 2006 were enrolled. This cohort is the same as the previous study of our group^40^, and this study has been performed independently of previous study. Samples from 2 patients had poor RNA quality and experiments were failed in these samples. Finally, analyses were performed in remaining 289 patients. Curative resection was defined as complete resection of all tumor nodules without involvement of microscopic resection margins and no detected residual tumor on computed tomography scans at 1 month after surgery. Twenty normal liver samples, which were confirmed biochemically and histologically and mostly obtained from patients with liver resection due to metastatic colorectal cancer, were selected as normal controls. The Institutional Review Board of Samsung Medical Center approved this study (2016–11–098), and waived informed consent. All research was performed in accordance with relevant guidelines/regulations.

By reviewing the medical records, clinical parameters such as age, gender, date of surgery, serum α-fetoprotein (AFP), and serum albumin were collected. HCC was diagnosed by basically histological feature by HE slide which shows hepatocytic differentiation, occasionally with aid of immunohistochemical staining of Hepatocyte antigen, AFP, or cytokeratin 19 when differentiation is poor. Histopathologic features of HCCs, including tumor differentiation, microvascular invasion, major portal vein invasion, intrahepatic metastasis, multicentric occurrence, and non-tumor liver pathology, were reviewed by two liver pathologists (S.Y.H. and C.-K.P.). Tumor differentiation was determined according to the criteria of Edmondson and Steiner^41^. Intrahepatic metastasis and multicentric occurrence were distinguished according to the criteria of the Liver Cancer Study Group of Japan^42^. Patients were staged according to the AJCC staging system^43^ and BCLC staging classification^44^.

During the follow-up period, patients underwent computed tomography (CT) with measurement of serum AFP every 2 or 3 months postoperatively. Patients with suspicious imaging findings and/or continuously elevated AFP levels were further evaluated with PET-CT and/or MRI. The median follow-up period was 119.8 months (range 14–151.4 months) for survival. Recurrence was generally diagnosed using radiologic examinations with no histologic confirmation. The site of first recurrence was classified as intrahepatic recurrence (IHR) or distant metastases (DM). IHR is defined as recurrence occurring anywhere inside the entire liver. Intrahepatic HCC recurrence within the first two years after curative resection is mainly due to intrahepatic metastasis, whereas late recurrence usually results from multicentric disease^45^. Using 2 years as a cutoff, intrahepatic tumor recurrence was classified as either early or late^46^. All other sites of recurrence were defined as DM. The duration of IHR-free survival (IHRFS), DM-free survival (DMFS), and recurrence-free survival (RFS) was calculated from the date of surgical resection to the date of each *event or the last day of follow-up*.

### RNA extraction and quantitative Reverse-Transcriptase Polymerase Chain Reaction (qRT-PCR)

Total RNA was isolated from sliced tissue samples using the RNeasy Plus Mini Kit (Qiagen, Hilden, Germany). RT-PCR was conducted using the High Capacity cDNA Reverse Transcription Kit (Applied Biosystems) according to the manufacturer’s instructions. Quantitation of miR-122 was performed using standard reagents from Applied Biosystems (TaqMan® MicroRNA Assays: miR-122: 002245). RNU6B was used as an endogenous control. Reverse transcription was performed with 25 ng of total RNA using the TaqMan® primers from MicroRNA Assays and the TaqMan® MicroRNA Reverse Transcription Kit (4366596, Life Technologies, Carlsbad). PCR was performed in an ABI PRISM 7500HT Fast Real-time PCR with TaqMan® Universal PCR Master Mix, No AmpErase UNG (Life Technologies) according to the manufacturer’s protocol. The PCR conditions were as follows: 95 °C for 10 min, followed by 40 cycles of amplification at 95 °C for 15 s and 60 °C for 1 min on the ABI PRISM 7500HT Fast Real-time PCR system (Applied Biosystems). The threshold cycle (Ct), which is the fractional cycle number at which the amount of amplified target reaches a fixed threshold, was determined. Relative changes in gene expression were measured using the 2−ΔΔCt (ΔΔCt = ΔCttarget gene−ΔCtGAPDH) method. Each reaction was carried out in triplicate technical replicates. Ct values were calculated for each replicate and averaged.

To generate standard curves for genotype identification assays, 10-fold serial dilution containing a HCC190 sample was analyzed in three independent runs by RT-qPCR. In order to take into account the diagnostic context of the sample matrix, each dilution was analyzed in three independent runs by RT-qPCR. The log dilution series of was tested in triplicates in each run. Standard curves for each assay were generated by plotting threshold cycle (Ct) values per three replicates per standard dilution versus the log concentration. Amplification efficiency, intra-assay and inter-assay variation were determined using 10-fold dilutions of one sample (No. 178). An efficiency of 1 corresponds to 100% amplification efficiency. The coefficient of determination (R^2^) was assessed and considered to be suitable when it was not lower than 0.98 in a single run.

### Statistical analysis

The cut-off value of miR-122 expression for RFS was determined using the log-rank test and the minimum p-value approach. The estimated cutoff value was internally validated using 1,000 bootstrap samples generated from the study data with replacement. *P*-values < 0.05 were considered statistically significant in two-tailed tests.

The correlation between miR-122 expression and other clinicopathologic variables was evaluated using the chi-square test or Fisher’s exact test. Univariate analysis was performed using the Cox proportional hazard model or the time-dependent Cox model according to satisfaction of the proportional hazard assumption for each variable. The proportional hazard assumption was confirmed using the correlation between partial residuals from the estimated Cox proportional hazard model and time to the event. Variables with *P* < 0.2 in univariate analysis were included in the multivariate analysis. Multivariate analysis was conducted using a time-dependent Cox model because some variables included in the multivariate analysis failed to adhere to the proportional hazard assumption. Multicollinearity was identified by a Variance Inflation Factor (VIF) >10.0. Some of those variables were excluded from the multivariate analysis. Statistical analyses were performed using SAS version 9.4 (SAS Institute, Cary, NC) and SPSS software (SPSS Inc., Chicago, IL, USA). Variable risk was expressed as a hazard ratio (HR) with corresponding 95% confidence interval (CI).

## Supplementary information

Supplementary Figure S1

Supplementary dataset 1
