# Supplementary material for: Prognostic significance of miR-122 expression after curative resection in patients with hepatocellular carcinoma
Source: Sci Rep. 2019 Oct 14;9:14738. doi: 10.1038/s41598-019-50594-2 (PMC6791887; doi:10.1038/s41598-019-50594-2)
Supplement: Supplementary file 1 — Supplementary Figure S1 [file 41598_2019_50594_MOESM1_ESM.docx]

**Prognostic significance of miR-122 expression after curative resection in patients with hepatocellular carcinoma**

Sang Yun Ha^1^, Jeong Il Yu^2^, Changhoon Choi^2^, So Young Kang^1,^ Jae-Won Joh^3^, Seung Woon Paik^4^, Seonwoo Kim^5^, Minji Kim^5^, Hee Chul Park^2*^, Cheol-Keun Park^1,6*^

^1^Department of Pathology and Translational Genomics, Samsung Medical Center, Sungkyunkwan University School of Medicine, Seoul, Korea,

^2^Department of Radiation Oncology, Samsung Medical Center, Sungkyunkwan University School of Medicine, Seoul, Korea,

^3^Department of Surgery, Samsung Medical Center, Sungkyunkwan University School of Medicine, Seoul, Korea,

^4^Department of Internal Medicine, Samsung Medical Center, Sungkyunkwan University School of Medicine, Seoul, Korea,

^5^Statistics and Data Center, Samsung Medical Center, Sungkyunkwan University School of Medicine, Seoul, Korea,

^6^Department of Pathology, Anatomic Pathology Reference Lab, Seegene Medical Foundation, Seoul, Korea

**Correspondence**

Cheol-Keun Park, MD, PhD. ckpark@mf.seegene.com

Hee Chul Park, MD, PhD. [hee.ro.park@samsung.com](mailto:hee.ro.park@samsung.com)

**Supplementary information:**

**
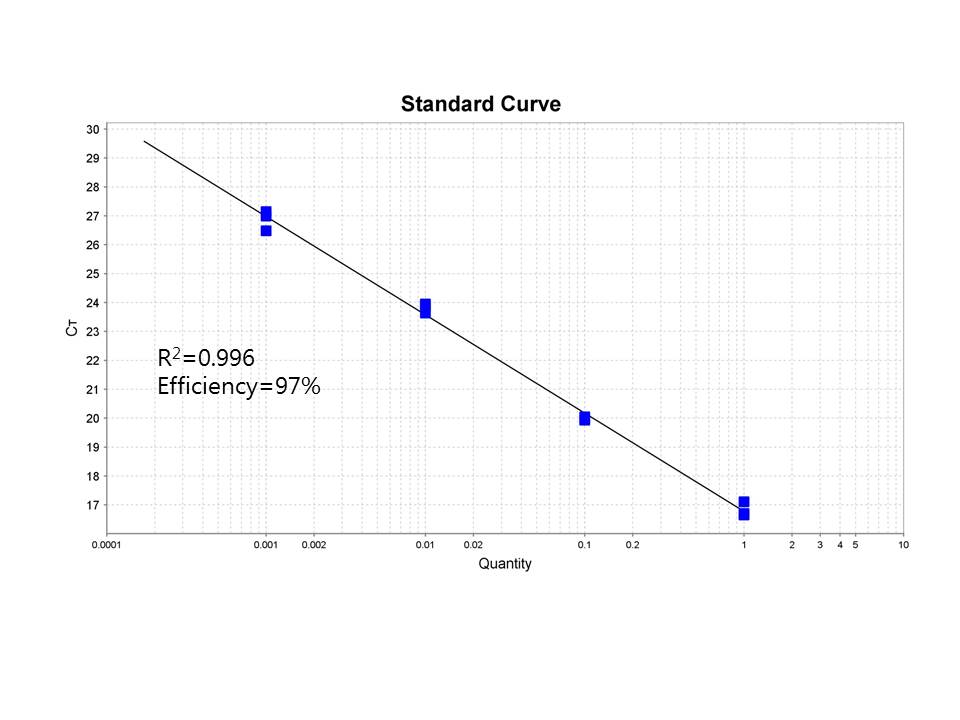
**

**Supplementary figure S1. Linear relationship between Ct values and log concentration of HCC sample**. The regression curve between Ct values and log concentration for serially diluted serum showed a good linear relationship (R^2^ = 0.992~0.996) when the Ct values were between 17 and 27. Serial tenfold dilutions of RNA were performed and miR122 expression was measured in these samples. The concentration of primary sample was arbitrarily designated as 1, and thus was 0.1 when the serum was diluted tenfold, etc. Each point represents the mean of triplicate measurements
